# What can reaction databases teach us about Buchwald–Hartwig cross-couplings?†

**DOI:** 10.1039/d0sc04074f

**Published:** 2020-10-20

**Authors:** Martin Fitzner, Georg Wuitschik, Raffael J. Koller, Jean-Michel Adam, Torsten Schindler, Jean-Louis Reymond

**Affiliations:** Roche Pharma Research and Early Development, pRED Informatics, Roche Innovation Center Basel, F. Hoffmann-La Roche Ltd Grenzacherstrasse 124 CH-4070 Basel Switzerland mart.fitzner@gmail.com; Roche Pharma Research and Early Development, pCMC Drug Substance, Roche Innovation Center Basel, F. Hoffmann-La Roche Ltd Grenzacherstrasse 124 CH-4070 Basel Switzerland georg.wuitschik@roche.com; Department of Chemistry and Biochemistry, University of Bern Freiestrasse 3 3012 Bern Switzerland

## Abstract

Despite the widespread and increasing usage of Pd-catalyzed C–N cross couplings, finding good conditions for these reactions can be challenging. Practitioners mostly rely on few methodology studies or anecdotal experience. This is surprising, since the advent of data-driven experimentation and the large amount of knowledge in databases allow for data-driven insight. In this work, we address this by analyzing more than 62 000 Buchwald–Hartwig couplings gathered from CAS, Reaxys and the USPTO. Our meta-analysis of the reaction performance generates data-driven cheatsheets for reaction condition recommendation. It also provides an interactive tool to find rarer ligands with optimal performance regarding user-selected substrate properties. With this we give practitioners promising starting points. Furthermore, we study bias and diversity in the literature and summarize the current state of the reaction data, including its pitfalls. Hence, this work will also be useful for future data-driven developments such as the optimization of reaction conditions *via* machine learning.

## Abstract 2

An analysis of the entire literature on Pd-catalyzed C–N couplings enables data-driven insight and provides recommendations for reaction conditions.

## Graphical abstract

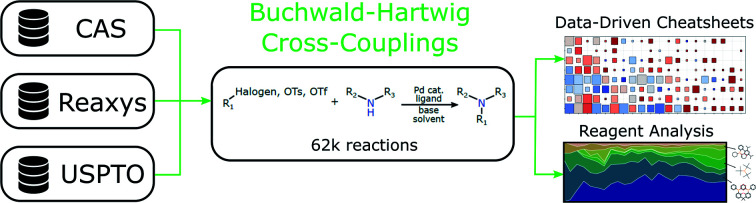

## 1. Introduction

Since its discovery,^1–5^ the Pd-catalyzed cross coupling of amines with aryl halides or pseudohalides has become widely used, due to its versatility and the importance of the products in many areas of applied chemistry. In a large variety of contexts, from the formation of heterocycles^6^ to the preparation of natural products^7^ to the synthesis of ligands,^8^ C–N bond formations are applied, and the number of applications for the resulting products are growing.^9^ Buchwald–Hartwig (BH) couplings are also an important tool in the pharmaceutical industry. They allow researchers to quickly assemble complex molecules and to graft nitrogen-containing functionality onto molecules.

Much has been learned about the mechanistic details of the underlying catalytic process over the past decades^10^ and chemically informed guidelines have been proposed.^9,11^ However, identifying suitable reaction conditions and then optimizing a given C–N coupling reaction can remain time-consuming. Despite the broad adoption of high-throughput experimentation^12^ in recent years, the large number of possibilities precludes exhaustive screening of reaction space. Self-guiding experiments^13^ can help, but the value of such experiments can be limited when the response curves are steep, as they are often for catalytic reactions. To address this challenge, we are seeing the advent of data-driven and/or machine learning methodology, which promises to use vast amount of existing reaction data to predict viable reaction conditions for a given set of substrates.

For instance, Ahnemann *et al.*^14^ created a yield-predicting model for the coupling of aryl halides with 4-methylaniline in the presence of different additives, where the data came from high-throughput experimentation. Sather and Martinot^12^ found through high-throughput experimentation working reaction conditions for piperidine-based nucleophiles with five-membered hetero-aromatic bromides, a known difficult class of BH substrates. Similar cases exist for other reactions such as deoxyfluorinations^15^ or thiol additions^16^ and more recently, Sandfort *et al.* have reported an approach based on fingerprints performing well at various chemical prediction tasks.^17,18^ Li and Eastgate developed a fingerprint based deep learning model that predicts the success probability of ligands for a given reaction, which was also used for insight on sustainability.^46^ For insight from the visual perspective, chemical informer libraries were developed to explore synthetic methods in complex structural space, such as Pd and Cu catalyzed couplings.^19^

In this work, we perform an extensive meta-analysis of the reaction landscape for C–N couplings of the BH type. We discuss trends and findings uncovered by analyzing more than 62 000 (∼46 500 with yield reported) unique BH couplings extracted from the CAS content collection,^20^ Reaxys®^21,22^ and the US Patent and Trademark Office (USPTO)^23^ databases.^24^ We aim to provide practitioners with solid starting points that are suitable for further optimization. We see this as an augmentation to the traditionally applied database searches or standard recipes. In addition, we also report some robust, overall trends in the data.

In Section 2.1 we introduce our data-pipeline, followed by a description of the reactant classifications we employ in Section 2.2. In Section 2.3 the reaction outcomes for different electrophile and nucleophile types are discussed. Section 2.4 provides the reagent recommendations resulting from this analysis, with a special emphasis on our ligand recommender in Section 2.5. Section 2.6 presents our analysis of the data diversity and time evolution. We conclude in Section 3.

## 2. Results and discussion

### 2.1 Reaction data processing

**Fig. 1:**
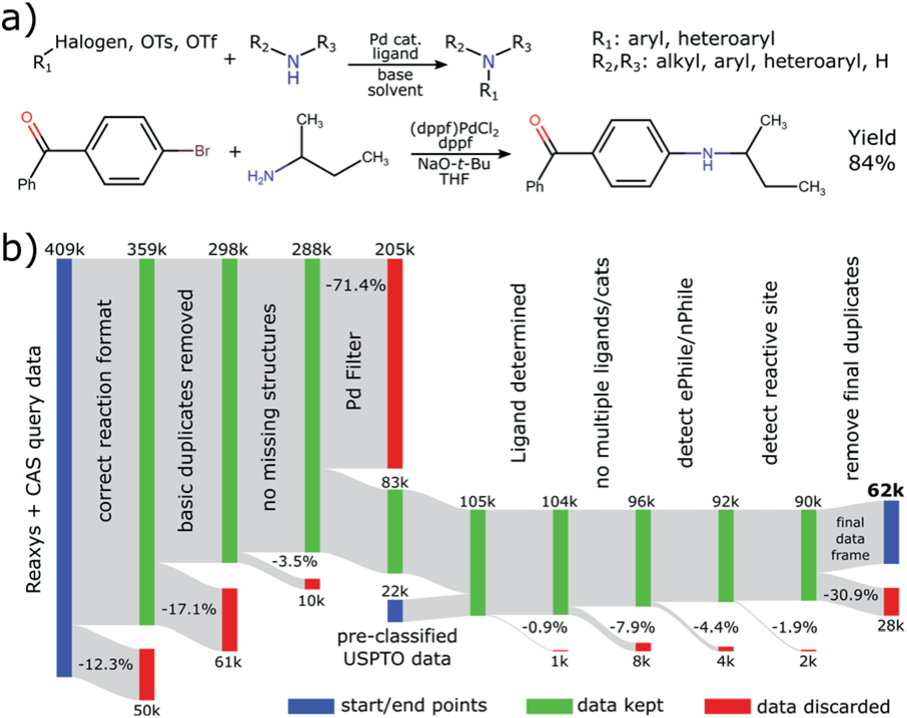
(a) Definition of the reaction studied in this work, together with an example from ref. 45 below. (b) Flow diagram outlining the data processing pipeline devised in this work.

To amass a substantial amount of reaction data suitable for the data-driven trend study applied in this work, we have conjoined data from three main sources: Elsevier's Reaxys, Chemical Abstract Services' SciFinder and patent data from the USPTO. Reactions from the latter have been extracted and made freely available^25^ while the other two are commercial providers of reaction and substance data. We provide visuals of our database queries in the ESI.† In Fig. 1a we show the definition and an example of a BH coupling as considered in this work.

Even though many steps were undertaken by the data providers to ensure a clean transition of the data from literature into their databases, there are still various normalization steps that we needed to conduct. An overview of our entire data processing pipeline and the discarded reactions in each step can be found in Fig. 1b.

As a first filter, we considered only reactions that have the coupling clearly identified as a single step and conform to a template combining two reactants into a single product (12.3% discarded) without missing reactant structures (3.5% discarded). The majority of reactions (75.0%) were conducted with Pd-based catalysts. Even though Cu-catalyzed Ullmann-type-couplings are 24.0% of C–N coupling reactions and Ni-catalysis has been employed more recently,^26^ we chose to focus our efforts on reactions with Pd. Many reactions are discarded by the transition-metal filter (71.4%) because the initial queries were deliberately designed to be as broad as possible, thus possibly also covering reactions that do not classify as BH coupling, for instance nucleophilic aromatic substitutions.

The need for additional data normalization is most striking for the reagents. Ligands and bases are not found in a corresponding data field, but rather in the generic reagents field. While solvents do have a dedicated data field, we find that they are still often declared as generic reagents. As a result, there is a large overlap and misclassification among the different reagent classes, see the ESI for further analysis.†

To correct this classification we introduce an array of cleaning steps, followed by table-lookup for solvents/bases and rule-based identification of ligands. The rules for the latter simply state that any reagent containing phosphorus, but no P–X bonds (to avoid reactive species) is a ligand. This rule is tested after looking up whether the substance is considered a base, to avoid phosphorus-containing bases being classified as ligands. We added additional rules to find N-heterocyclic carbene (NHC) ligands *via* simple substructure searches. Any unrecognized substance is then categorized as generic reagent, which also allows for iterative updates of our lookup tables and rules.

We find that the ligand is often given either as a mixture with a Pd salt or as a defined Pd complex. Thus, another step added is to sever bonds between phosphorus and the transition metal to extract the ligand. In some cases (0.9%) we are not able to detect the ligand and in others (7.9%) we find multiple possible ligands. These reactions were discarded because we cannot reliably determine which is the active species. In addition, while the form of pre-catalyst can be important for reaction performance, we decided to subsume them under the respective ligands employed in order to reduce the number of catalyst species for analysis. We find that the most commonly used pre-catalysts are Pd_2_(dba)_3_ (55%) and Pd(OAc)_2_ (29%) with G pre-catalysts having surprisingly little uptake (4%). Further details can be found in the ESI.† After this stage, we perform various molecular cleaning steps to normalize structures.^27^

Lastly, we address the need to remove duplicated reactions. A first duplicate removal is done in the beginning of the pipeline by considering the raw data. This is referenced as “removing basic duplicates” in Fig. 1b. Another round of identifying duplicates is necessary as very last step after all reagent types have been assigned. This is because there could be overlap between the various data sources and some reactions appear multiple times, for example in several patents. The identification is done by concatenating and hashing the canonical representation strings (InChI) of all relevant molecules in a fixed order to obtain a reaction key. In this manner we identify all reactions as identical that use the same electrophile, nucleophile, product, solvent, base and ligand. This would likely fail if it were not for the previously discussed cleaning steps. Even though other parameters like temperature, reaction time or scale could differentiate these, we find that the yield reported for a set of reactions flagged as identical is mostly the same (other than for rounding errors), indicating that indeed identical reactions were detected. For each set of duplicated reactions, we only keep the entry that was published first, to gain an overview of novel entries over time. According to these criteria, 30.9% of reactions were identified as duplicates by the second deduplication.

After all steps we obtain 62 011 cleaned and unique BH reactions, 46 527 (75.0%) of which have a yield reported. We note that the availability of other relevant metadata is lower (temperature 59.2%, reaction time 66.7%) and not always precise (*e.g.* “overnight” given for reaction time). We also note that these data do not allow for a good estimate of the reaction scale or the catalyst loading, even though these parameters could influence reaction performance.

### 2.2 Reactant classification

We now introduce a classification of the two reactants. First, we detect which reactant is the electrophile and which is the nucleophile. If the sum of leaving groups in the reactants is exactly one more than in the product for a certain type of leaving group (we considered Br, Cl, I, F, OTf and OTs) then the electrophile is the reactant which has one or more of that leaving group. This fails if both reactants have that leaving group, if the change in leaving group count is more than one or if more than one type of leaving group is changing. In these cases we analyze the count of nitrogen atoms with an attached H to identify the nucleophile. If this also fails, we cannot identify the electro-/nucleophile and discard these reactions (4.4%).

To obtain a description of the reaction site we need the indices of the reactive carbon and nitrogen atoms on the electrophile and nucleophile respectively. This is straightforward with reaction mapping. However, we find that this information can be erroneous and is not present in all of the data. We have therefore devised a method to identify the reacting atom of each reactant automatically, customized for this kind of reaction. For the electrophile, this includes severing all bonds between leaving groups and carbon atoms, and checking whether the remaining molecule is a substructure of the product. If this is true for exactly one leaving group tested, the carbon atom connected to that group is the reactive one. For the nucleophile, this involves a similar procedure, replacing one of the hydrogen atoms connected to all possible nitrogen atoms by a carbon and checking whether the resulting molecule is a substructure of the product. In some rare cases, the tautomeric form of the product is different from the reactants, which prevents this algorithm from working (*e.g.* hydroxypyridine and pyridone). To accommodate this, we are iterating through all tautomers of the product to check if the relevant substructures can be found in any of them, which still leads to a unique result in almost all cases. This overall method succeeds for 98.1% of reactions, yielding the possibility to classify the surrounding of the reacting nitrogen and carbon.

For electrophiles we employ a simple classification by leaving group and whether it is attached to an aryl (ARY) or heteroaryl (HAR). For the nucleophile we consider several classes that correspond to the different bonding environments possible. If the reacting nitrogen is connected to one/two aliphatic carbons, we name the nucleophile as Alkyl/DiAlkyl. Likewise, if it is connected to one/two aromatic atoms it is named Aryl/DiAryl and Alkyl–Aryl if the nitrogen is connected to both one aliphatic and one aromatic carbon. If the nitrogen is part of an aromatic system it is classified as aromN and if it exclusively has one double bond to a carbon it is named Ketimine. As a special case, we are also detecting amides *via* substructure search.

With this reactant classification, we are able to achieve a more fine-grained overview of the reaction performance of the various reagents, depending on electrophile and nucleophile classes. We show examples for the various nucleophile classes in the ESI.†

### 2.3 Nucleophile/electrophile yield trends

**Fig. 2:**
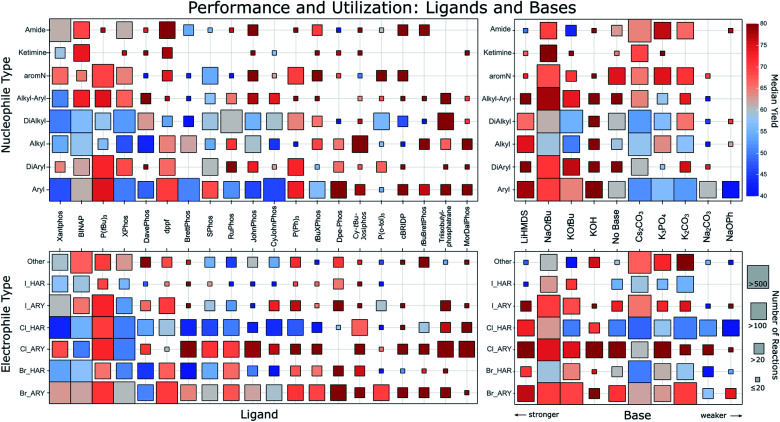
Ligand (left column) and base (right column) performance for different nucleophile types (top row) and electrophile types (bottom row). The order of the ligands goes from most common (left) to lesser common (right) and bases are ordered from strong to weak. Only the top 20 ligands and top 10 bases are shown. The different nucleophile types describe the surrounding of the reacting nitrogen. Aryl: single aromatic C bonded to N; DiAryl: two aromatic C’s bonded to N; Alkyl: single aliphatic C bonded to N; DiAlkyl: two aliphatic C’s bonded to N; Alkyl–Aryl: one aromatic and one aliphatic C bonded to N; aromN: N part of an aromatic ring; Ketimine: aliphatic C connected with a double bond to the N. Electrophiles are characterized by their leaving group and whether it is attached to an aryl (ARY) or heteroaryl (HAR). The square color corresponds to the median reported yield for all reactions falling into the category. The square size corresponds to the amount of reactions as indicated on the right. An interactive version of this figure is part of the ESI.†

Fig. 2 squares the nucleophile and electrophile classes with the most commonly found ligands and bases. For each intersection, the number of reactions and their median yield is represented by a square of matching size and color, revealing large differences. Before discussing the trends visible therein, we point out that an interactive version of this figure is part of the ESI for the reader to explore.†

Only a few ligands have been used for all types of nucleophiles, and so far no ligand has emerged that results in universally high yields. The performance across all nucleophile types seems better for ligands used less frequently. Favorites are visible for some substrate classes, for instance Dpe-Phos for aryl amines, Cy-^*t*^Bu-Josiphos for alkylamines or triisobutylphosphatrane for dialkylamines. The most popular of all ligands, Xantphos displays a comparatively low average yield. This may be the result of Xantphos' low cost and broad substrate scope, potentially making it a first-line ligand for applications in which yield is of secondary importance. A clear yield difference can also be observed in the electrophile category, in that more recently reported ligands like MorDalPhos or triisobutylphosphatrane show significantly higher yields than more frequently used ligands. Furthermore, it is clearly visible that in general, aryl chlorides have a much better performance compared to heteroaryl chlorides. To a lesser extent, this is also visible for bromides.

As expected, stronger bases are often employed for the arylation of weakly acidic alkylamines, with potassium hydroxide, mostly in water or tertiary alcohols, showing surprisingly good yields across a variety of substrates. Weaker bases are also often used for these amines, but the reported yields for reactions of weakly acidic amines with such bases are significantly lower than for more acidic amines.^28^ Their lower p*K*_a_ makes it harder to deprotonate the Pd-coordinated amine during the catalytic cycle.^11^ However, the lower yields may also be a consequence of higher substrate complexity and presence of base-labile functional groups for which stronger bases would perform worse.

It is important to consider biases in the literature that potentially influence the figures shown herein. For instance, recommendations based on only a few entries could stem from reactions that only utilized very simple substrates. This scenario is less likely for conditions backed up by many literature entries. The different degree of reaction optimization will also introduce bias. For example, one may expect combinations of ligands and bases that feature in standard recipes to be used more often in settings like medicinal chemistry, where yield optimization is not always a priority. As proxy for judging the diversity and simplicity, we show in the ESI† versions of the matrices from Fig. 2 plotting instead of the median yield one of these four quantities: (i) exact number of reactions; (ii) mean molecular weight of products; (iii) mean heteroatom count of products and (iv) mean Tanimoto distance between products. With this we are able to spot potentially problematic entries. Besides the biases we already discussed we note that for ligands P(^*t*^Bu)_3_, dppf, P(*o*-tol)_3_ and Triisobutylphosphatrane the reactions seem to show slightly lower difficulty and diversity. For bases we find in particular that KOH was used on a set of easier and less diverse substrates compared to other bases.

### 2.4 Reagent recommendation

**Fig. 3:**
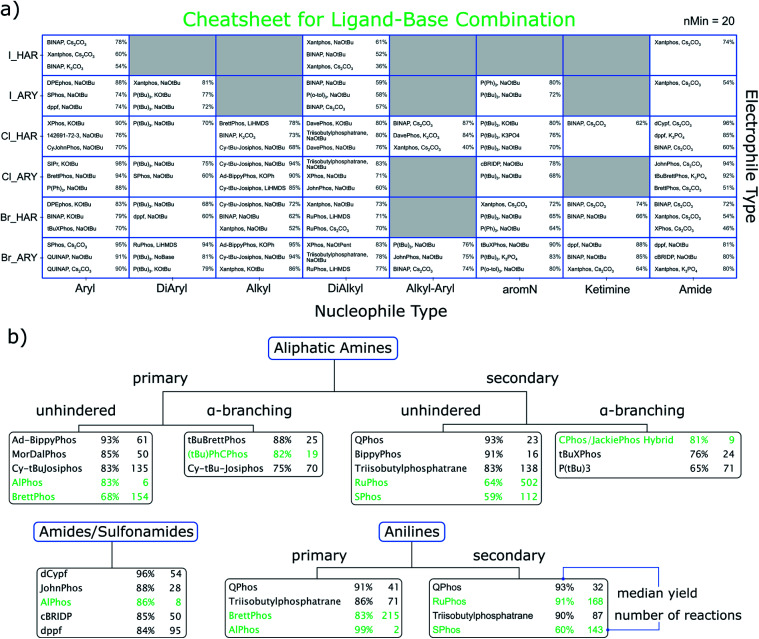
(a) Cheatsheet for data-based recommendation of ligand-base combinations as a function of the electrophile type (rows) and nucleophile type (columns). Shown are the three conditions that had the highest median yield, provided at least 20 reactions were in the data for that combination. Numbers on the right side of a tile are the median yields for the conditions to the left. The ligand with the CAS number 142691-72-3 is 1,1′-bis[bis(dimethylamino)phosphino]ferrocene. (b) Our perspective of the cheatsheet by Ingoglia *et al.*^11^ Highlighted in green are their suggestions and how they rank in our data set. The black entries represent our suggestions, which were made by considering median yield, availability and number of reactions in our data. The complete tables behind the ranking are available in the ESI.†

With the nucleophile and electrophile classifications introduced, we are able to suggest promising sets of conditions for combinations. The result is displayed in Fig. 3a, where we show a cheatsheet for selecting the most promising ligand/base combination. The recommendation is made based on finding the top three ligand/base combinations ranked by median yield for each electrophile/nucleophile combination. Since these combinations are already very specific, the data available for each selection can be sparse. Thus, we only report a recommendation if there are at least 20 (ref. 29) reactions for it.^30^ As an example, for coupling a primary aniline (ARY) to a heteroaryl chloride (Cl_HAR) the top recommended ligand/base combination would be XPhos and KO^*t*^Bu with a literature-reported median yield of 90%.

In order to benchmark our results, we also adopted the classification scheme of Ingoglia *et al.*, which distinguishes nucleophiles into ten categories,^31^ also including steric hindrance.^11^ Based on median yield, commercial availability of the ligand and number of reactions, we chose three ligands each for aliphatic amines, anilines and amides for which sufficient hits were part of the dataset. These are displayed in Fig. 3b and show significant overlap (highlighted in green) with the previously published cheatsheet from ref. 11 and 32. In some cases our recommendation based on the data differs, in that higher yielding and commonly used alternative ligands are found.

For Fig. 3a we performed a similar diversity and difficulty analysis as mentioned for Fig. 2 (see ESI†). We find that most recommendations show comparable or better metrics as the ones reported in the original cheatsheet (green entries). The reader might also wonder about the errors of the reported yields in the cheatsheet. We have refrained from reporting those in here as they are generally large and should not be confused with prediction intervals (albeit we rank the relative performance by median yield, this should not be considered a yield prediction). Our interactive html files show the interquartile ranges of the reported yields for each recommendation.

### 2.5 Ligand recommender

**Fig. 4:**
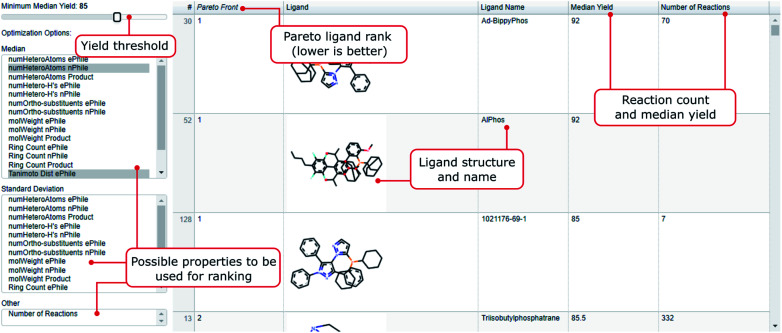
Explanation of the interactive ligand recommender that is part of this work: A Pareto ranking sorts all ligands in our dataset based on the properties selected by the user. Properties available are median molecular weight, number of heteroatoms, ring count, number of heteroatom-H substructures, number of ortho-substituents, yield and Tanimoto distance (based on Morgan fingerprints of radius 4 (ref. 43 and 44)) for the reactants and/or the product. If the user is interested in ranking ligands by the diversity of their application, they can also choose the standard deviation of these properties instead of the median. After the ranking the user can exclude ligands below a selected median yield (slider top left). After that, the remaining ranked ligands are promising candidates for the reactant properties that were selected. This tool does not discriminate against the number of reactions used and thus is promising to discover more rarely used yet promising ligands.

As a step towards improving the recognition of ligands that are less frequently used but could be valuable, we propose the tool highlighted in Fig. 4. In there, the user can sort ligands according to multiple properties like molecular weight, heteroatom or ring count of the reactants and products used in conjunction with the ligand.^33^ In the resulting list, ligands are sorted according to the parameters specified by the user as the result of a Pareto ranking.^34^ The rank assigned is called the Pareto front. A ligand or set of ligands would be called Pareto-optimal, if all other ligands are worse in at least one of the selected properties. The next layer of ligands are assigned rank two, and so on. The user can also choose to only include ligands with a median yield above a threshold, or incorporate either median yield or number of reactions in the Pareto ranking itself.

The tool is generally used in two steps. First, the user selects the properties on the left according to which they wish to order the ligands. The ligands with the highest of these values will be ranked (rank 1 being best). After that the user is able to adjust the yield slider on top. Ligands which have a median yield lower than the selected value will not appear in the list, even if they were ranked high. This serves the purpose of filtering from the ranked ligands the ones which performed well. By inspecting the number of reactions for an entry the user can judge whether they take this recommendation or want a safer recommendation with more literature entries backing it up (by *e.g.* lowering the yield slider). In the following we provide three use cases as examples of how to use the tool:

1. The user wants to have the best ligand for a scenario where both ePhile and nPhile have a lot of heteroatoms. They would select both the median_numHeteroAtoms_ePhile and median_numHeteroAtoms_nPhile on the left. Assuming that the yield slider is at 95% the two most promising ligands would be meCgPPh and CAS 2144425-53-4. However, the number of reactions for these ligands is just 37 and 6 respectively. If the user wants a less risky recommendation they could shift the ligand slider to lower values. For instance, at 80% we have Ad-BippyPhos and dCypf as most recommended ligands with reaction numbers of 70 and 67 backing this up.

2. The user has a sterically hindered electrophile. They would select median_numOrtho-substituents_ePhile and have TNpP as the best ranked ligand amongst ligands with median yield above 85%.

3. Suppose the user is not interested in a recommendation for one particular reaction but they would like to design a plate with ligands that can perform well on a variety of ePhile properties. Such diversity could be measured with the median Tanimoto distance. The user would select median_TanimotoDist_ePhile, ranking ligands that had a good variety in their electrophiles highest. Assuming the yield slider is at 80%, the most recommended ligand would be ^*t*^BuBrettPhos. It is also worth pointing out that ligands ranked lower (ranks 2 and higher) could also be very promising candidates.

With this tool, we allow the user to go beyond typical classification schemes and provide a means of finding infrequently used but promising ligands. This could for example improve the design of high-throughput experimentation plates. In the future, we hope to provide individualized recommendations based solely on the structure of reactants and product by employing machine learning.

### 2.6 Data diversity and time evolution

**Fig. 5:**
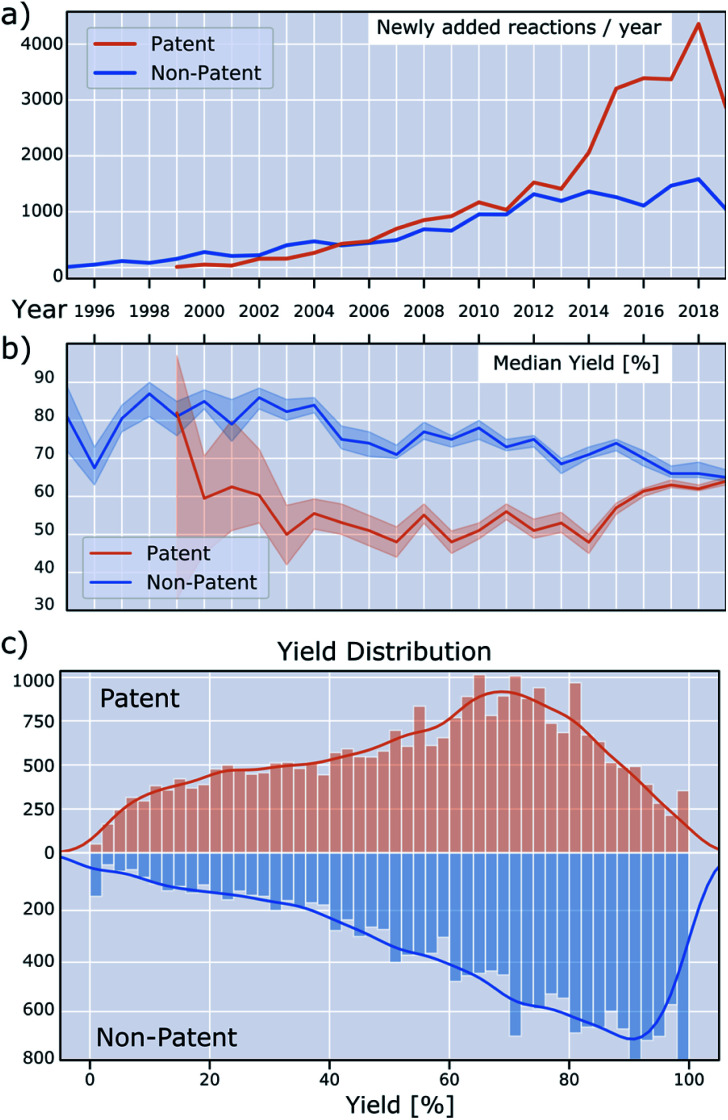
(a) Number of newly added reactions per year. (b) Median reported yield over time. The shaded lines are 95% bootstrapped confidence intervals for the yield median (not to be confused with the yield distribution quartiles, which are large and not shown). (c) Histogram of reaction yield from patents and non-patents.

Currently, at least 5000 new BH couplings are reported every year, as seen in Fig. 5a.^35^ The rising popularity of this reaction is evident from the number of reactions in both patent and non-patent literature. New reactions are increasing in both categories, with the number in the former outpacing the latter significantly since 2014. A similar change in trend around the same time is visible in the median reaction yield of the patent literature *versus* non-patent publications (Fig. 5b): Whereas the median yield in the patent literature was 20–30% lower before 2014, this gap has virtually disappeared since then. Overall, median yields in the non-patent literature trend downward over time, which may be the result of rising substrate complexity or increasing use in areas with lower emphasis on reaction optimization.

Fig. 5c shows the overall yield distribution, split into patents and non-patents. These data are noteworthy for several reasons. First, both the distribution in patent and non-patent reactions is skewed towards higher yields, possibly because lower-yielding reactions were optimized further and then only the optimized conditions were reported. This is well-known, but not ideal for data-driven approaches, such as machine learning. Ideally, the reaction yields should cover the reaction space as broadly as possible.

**Fig. 6:**
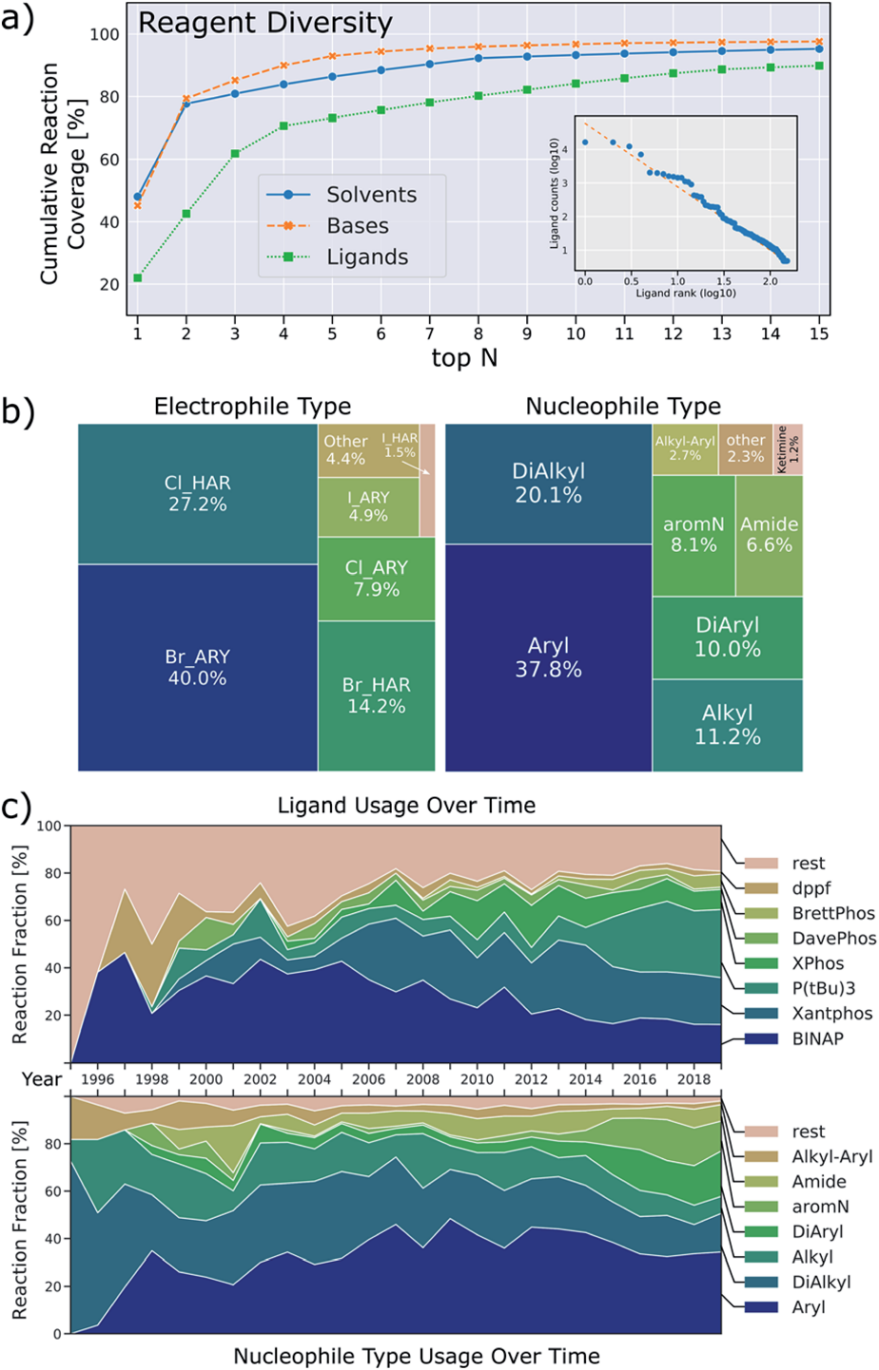
(a) Cumulative reaction coverage by the top solvents, bases and ligands. The inset shows the frequency-rank distribution of ligands (double logarithmic axes) which approximately follows Zipf's law. (b) Relative occurrence of electrophile types and nucleophile types used. (c) Relative occurrence of the overall most frequently used ligands (top) and nucleophile types (bottom) over time.

A second aspect of the data that can be detrimental to machine-learning applications is the low diversity with respect to the number of reagents utilized, depicted in Fig. 6a. For instance, 79% of reactions use either sodium *t*-butoxide or cesium carbonate as base and 78% of reactions use either toluene or dioxane as solvent. This means that only a relatively small set of data includes other bases and solvents, weakening the predictive power of a machine learning model that includes base/solvent parameters. This lack of data diversity is particularly pronounced for reagents, and it will be hard to devise sampling-schemes to remedy this. Ligand usage is slightly more diverse in that 80% of reactions use one of the top eight ligands. We also investigated Suzuki-couplings and, in contrast to BH couplings, the diversity of solvents is high, but the diversity of ligands is low (twelve binary solvent combinations and only two ligands needed to cover 80% of all reactions).^36^

As a third observation, for non-patent reactions, a remarkable pattern emerges in which the most common yields are whole-number multiples of ten, specifically for yields greater than 30%. This means that yields of 40, 50, 60, 70, 80 and 90% are reported more frequently than would be expected, resulting in spikes in the yield distribution. This irregularity is less pronounced for reactions found in patents and it is not clear what causes this discrepancy. The data providers confirm that this originates from the reported data, and is not an artifact of data collection and processing.

We note an additional peculiarity of the observed ligand distribution: the frequency of usage *versus* rank of usage for ligands resembles a Zipf distribution (inset in Fig. 6a).^37^ This means that the frequency of ligands is inversely proportional to their frequency rank. Zipf's law appears in a variety of fields: the frequency with which words are used follows this distribution for most languages^38^ as well as the population ranks of cities,^39^ firm sizes^40^ and neural activity.^41^ Based on attempts to rationalize these findings, we can only speculate as to what the origin of this observation might be. Human bias to first try ligands that are both familiar and available as well as limited resources will lead to some ligands being used more frequently. It may be that the forces that shape the resulting distribution are similar to the principle of least effort that was stipulated to result to the word frequencies observed in linguistics.^42^

Fig. 6c also shows how the distribution of nucleophiles and ligands has changed over time. While alkylamines dominated historically, most reported BH couplings are now on aromatic amines. Use of modern dialkylbiaryl phosphine ligands slowly increases, but older ligands like BINAP, Xantphos and tri-*t*-butylphosphine are still used predominantly. The data also show how difficult it is for new ligands to find widespread application. For the leaving group, aryl bromides are the most common electrophile, followed by heteroaryl chlorides/bromides, other leaving groups only play a minor role (Fig. 6b).

Around 2014, the data show a large increase in the usage of tri-*t*-butylphosphine as a ligand, and a rise of diarylamine and aromatic nitrogen nucleophiles (Fig. 6c). Inspection of the underlying patent literature confirms that all of these observations are caused by work executed to prepare polyaromatic compounds of interest to OLED-applications. Most of these reactions use tri-*t*-butylphosphine as ligand with sodium *t*-butoxide as base in an aromatic solvent. The typical yield of these reactions is higher than those for reactions of other nucleophiles, thus causing the median yield to increase around that time. This example illustrates how demand for certain product classes can skew the data. It is therefore important to consider substrate structure when drawing conclusions about the prevalence and performance of ligands, bases and solvents.

## 3. Conclusion

In this work we approached the problem of reaction condition optimization for BH couplings from a big-data perspective, employing a meta-analysis of data from the CAS content collection, Reaxys and the USPTO. After normalization and classification of the data into chemically intuitive categories for nucleophiles and electrophiles, we provide cheatsheets and recommendation tools to improve the selections of reagents. These tools were designed to be usable by the practitioner with minimal effort. Our results provide guidance for chemists, helping them to facilitate reaction condition selection on a sounder basis.

Our analysis of the data uncovered some aspects that influence interpretation: beyond a skewed yield there is a significant imbalance in the reagent diversity. There are a few frequently used favorites for solvents, bases and ligands. However, these preferences stem likely from availability, cost and historical reasons, and not necessarily from superior reaction outcomes. Based on this, exploring a wider range of ligands is generally recommended.

This work shows that additional effort is needed to overcome data-inherent bias and allow for substrate-specific predictions of reaction conditions. To enable future projects in the realm of machine learning, generating data points with more diverse reaction conditions should be prioritized over higher yields and it is vital that all rather than just the best conditions need to be reported. With the exponentially growing number of data points it will be interesting to reanalyze this data in a few years.

## Data availability

The raw data used for this study is licensed from CAS and Elsevier. The USPTO data can be found in ref. 25.

## Author contributions

J. M. A., T. S. and M. F. conceived the research project. M. F. performed the data acquisition, cleaning and the overall pipeline. G. W., R. J. K., T. S. and J. M. A. contributed to several chemistry aspects of the data pipeline. M. F. created the figures and interactive plots. M. F. and G. W. analyzed the data and wrote the initial draft. All authors contributed to the interpretation of the data and the writing of the publication.

## Conflicts of interest

The authors declare no competing financial interest.

## Supplementary Material

SC-011-D0SC04074F-s001

SC-011-D0SC04074F-s002

SC-011-D0SC04074F-s003

SC-011-D0SC04074F-s004

SC-011-D0SC04074F-s005

SC-011-D0SC04074F-s006

SC-011-D0SC04074F-s007
