# Supplementary material for: What can reaction databases teach us about Buchwald–Hartwig cross-couplings?
Source: Chem Sci. 2020 Oct 20;11(48):13085–93. doi: 10.1039/d0sc04074f (PMC8378852; doi:10.1039/d0sc04074f)
Supplement: SC-011-D0SC04074F-s005 [file SC-011-D0SC04074F-s005.html]

Reagent Matrix Explorer
