# Supplementary material for: What can reaction databases teach us about Buchwald–Hartwig cross-couplings?
Source: Chem Sci. 2020 Oct 20;11(48):13085–93. doi: 10.1039/d0sc04074f (PMC8378852; doi:10.1039/d0sc04074f)
Supplement: SC-011-D0SC04074F-s007 [file SC-011-D0SC04074F-s007.pdf]

Electronic Supplementary Material (ESI) for Chemical Science.
This journal is © The Royal Society of Chemistry 2020

Supporting Information for: What Can Reaction Databases Teach Us About Buchwald-Hartwig Cross-Couplings?

Martin Fitzner,*,† Georg Wuitschik,‡ Raffael J. Koller,‡ Jean-Michel Adam,‡ Torsten Schindler,† and Jean-Louis Reymond¶

†*Roche Pharma Research and Early Development, pRED Informatics, Roche Innovation Center Basel, F. Hoffmann-La Roche Ltd, Grenzacherstrasse 124, CH-4070 Basel, Switzerland*

‡*Roche Pharma Research and Early Development, pCMC Process Research, Roche Innovation Center Basel, F. Hoffmann-La Roche Ltd, Grenzacherstrasse 124, CH-4070 Basel, Switzerland*

¶*Department of Chemistry and Biochemistry, University of Bern, Freiestrasse 3, 3012 Bern, Switzerland*

E-mail: mart.fitzner@gmail.com

Contents

1 Details on Database Queries **3**

2 Data Normalization Sanity Check **4**

3 Zipf's law **5**

4 Substrate Classification Examples **6**

5 Additional Time Evolution Plots **7**

6 Reaction Diversity Analysis **9**

6.1 Augmented Cheat Sheet 9

6.2 Nucleophile Type vs. Ligand 10

6.3 Electrophile Type vs. Ligand 11

6.4 Nucleophile Type vs. Base 12

6.5 Electrophile Type vs. Base 13

7 Substance Utilization **14**

8 Pre-Catalyst Analysis **16**

9 More Cheatsheets **17**

10 Ligand Ranking by Nucleophile Type **18**

1 Details on Database Queries

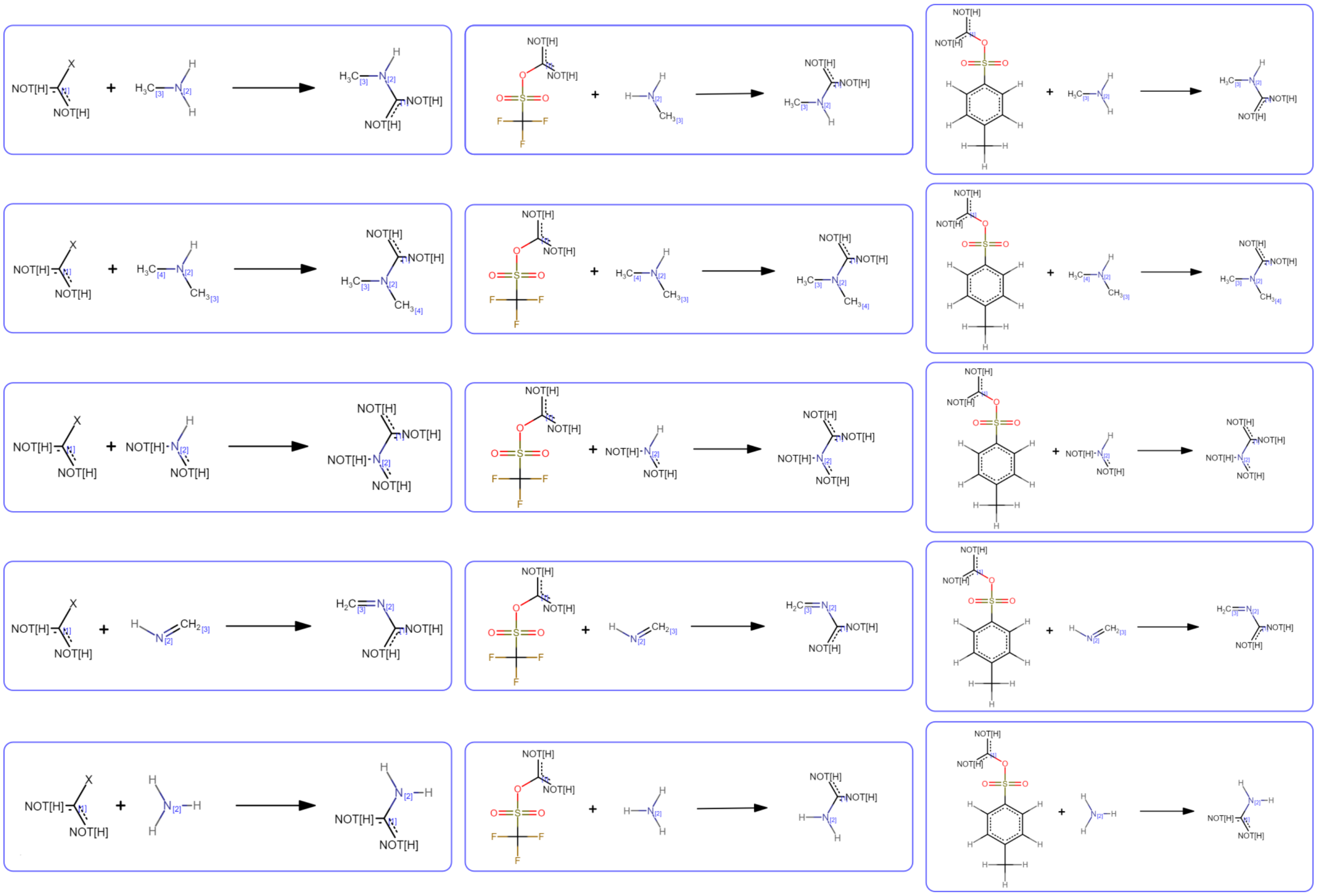

Figure S1: Visualization of the 15 queries that were posted to Reaxys and later on combined with the data from the other two data bases.

2 Data Normalization Sanity Check

We characterize the overlap between two sets X and Y of substances with the Szymkiewicz–Simpson coefficient, which gives a measure for how much of the smaller set is contained in the larger one:

$$\text{overlap}(X, Y) = \frac{|X \cap Y|}{\min(|X|, |Y|)}$$

We see that our normalization procedure gives a consistent result in that there is no overlap

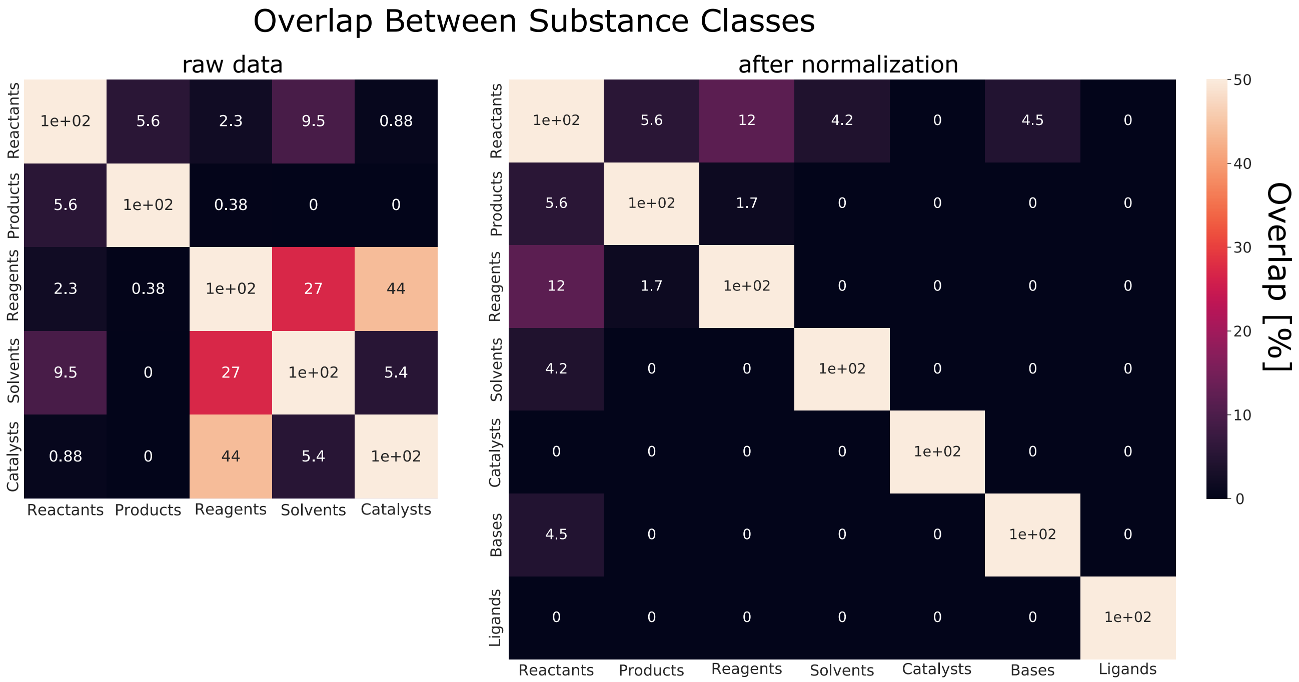

Figure S2: Overlap between the different substance classes. The matrix on the left corresponds to the raw data, where it is evident that no data fields for base or ligand are present. The matrix on the right corresponds to the result obtained after our normalization procedures. Colors and numbers correspond to the overlap value according to the color bar on the right.

between reagents left (lower right sub matrix). A residual overlap between certain reactants, products and generic reagents is expected.

3 Zipf's law

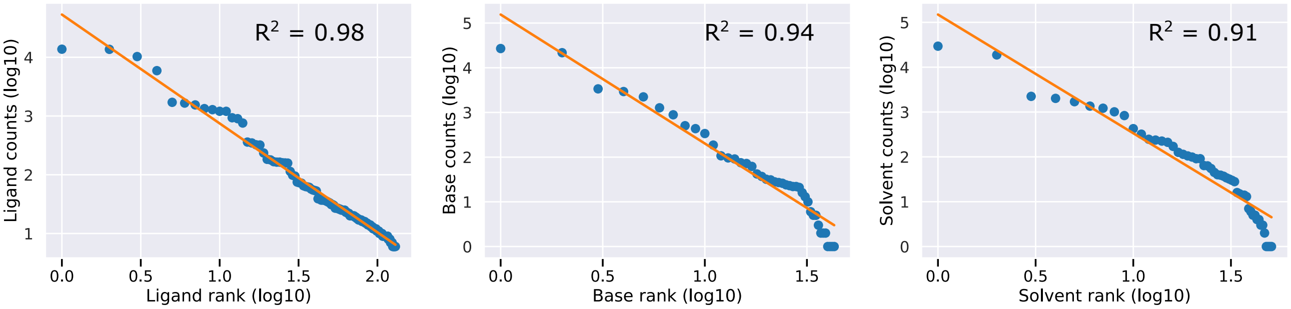

Figure S3: Log-log plots of the substance counts versus the substance rank (ranked by frequency of usage) for ligands, bases and solvents. We find that all three of these approximately follow a Zipf distribution, indicated by high R^2 values. Furthermore we performed a χ^2 goodness-of-fit test (on the untransformed data) and found no evidence to support the null hypothesis in any of them ($p \approx 1$).

4 Substrate Classification Examples

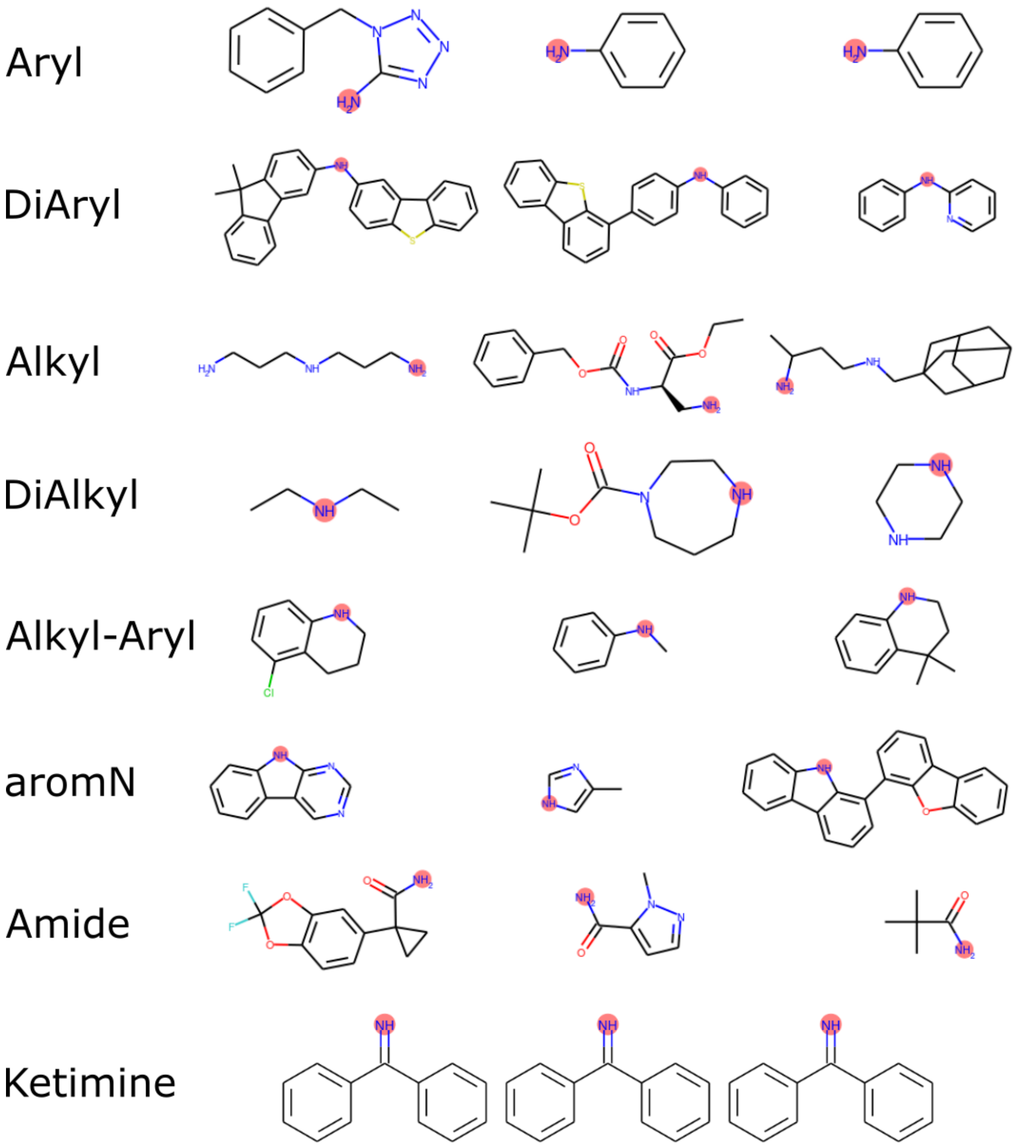

Figure S4: Three molecule examples (right) for each of the substrate classes (left) investigated in this work. The coupling nitrogen is highlighted in red. We note that for ketiminies we essentially only find one single molecule.

5 Additional Time Evolution Plots

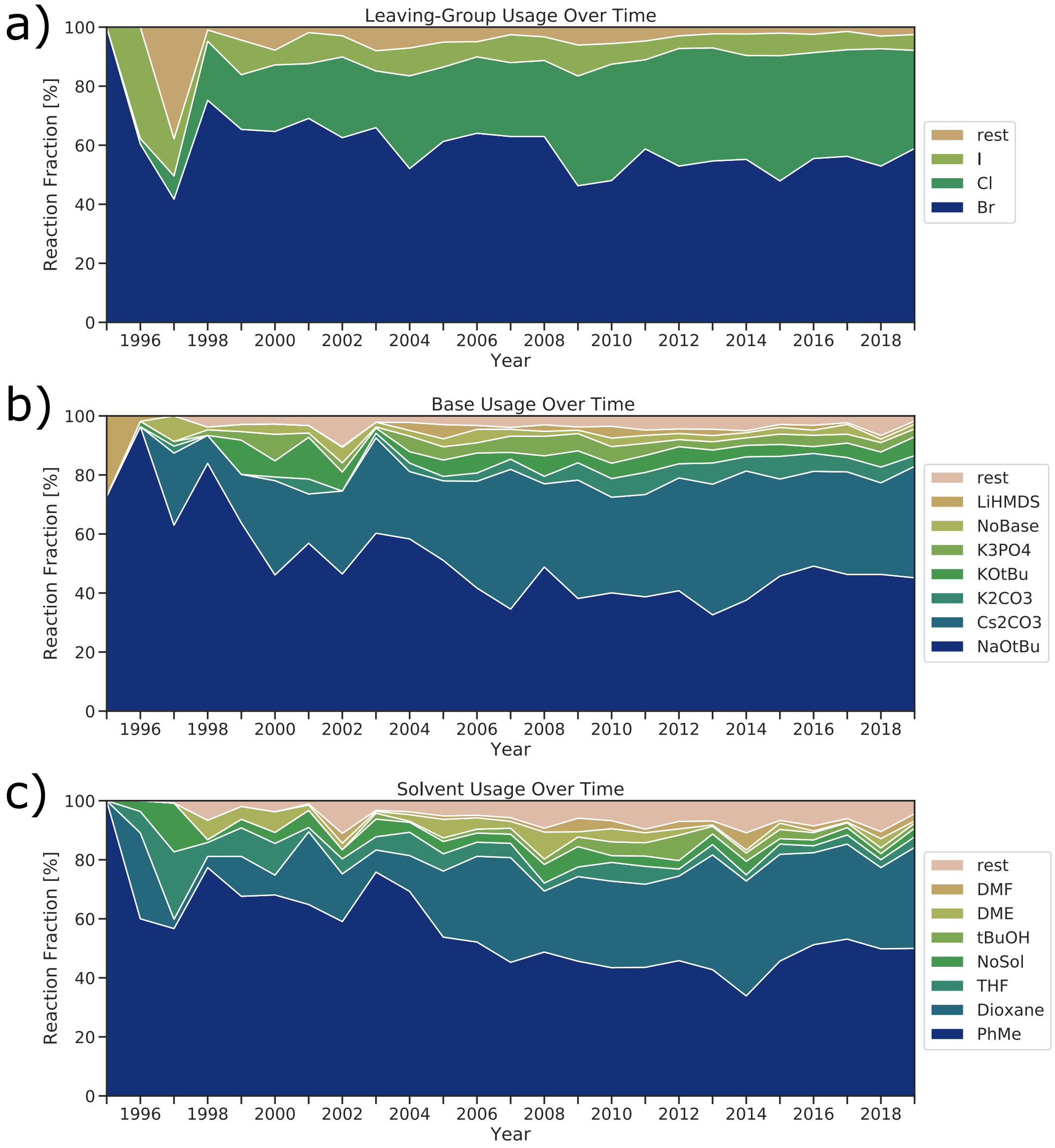

Figure S5: Time evolution of the leaving groups (a), bases (b) and solvents (c) used over time.

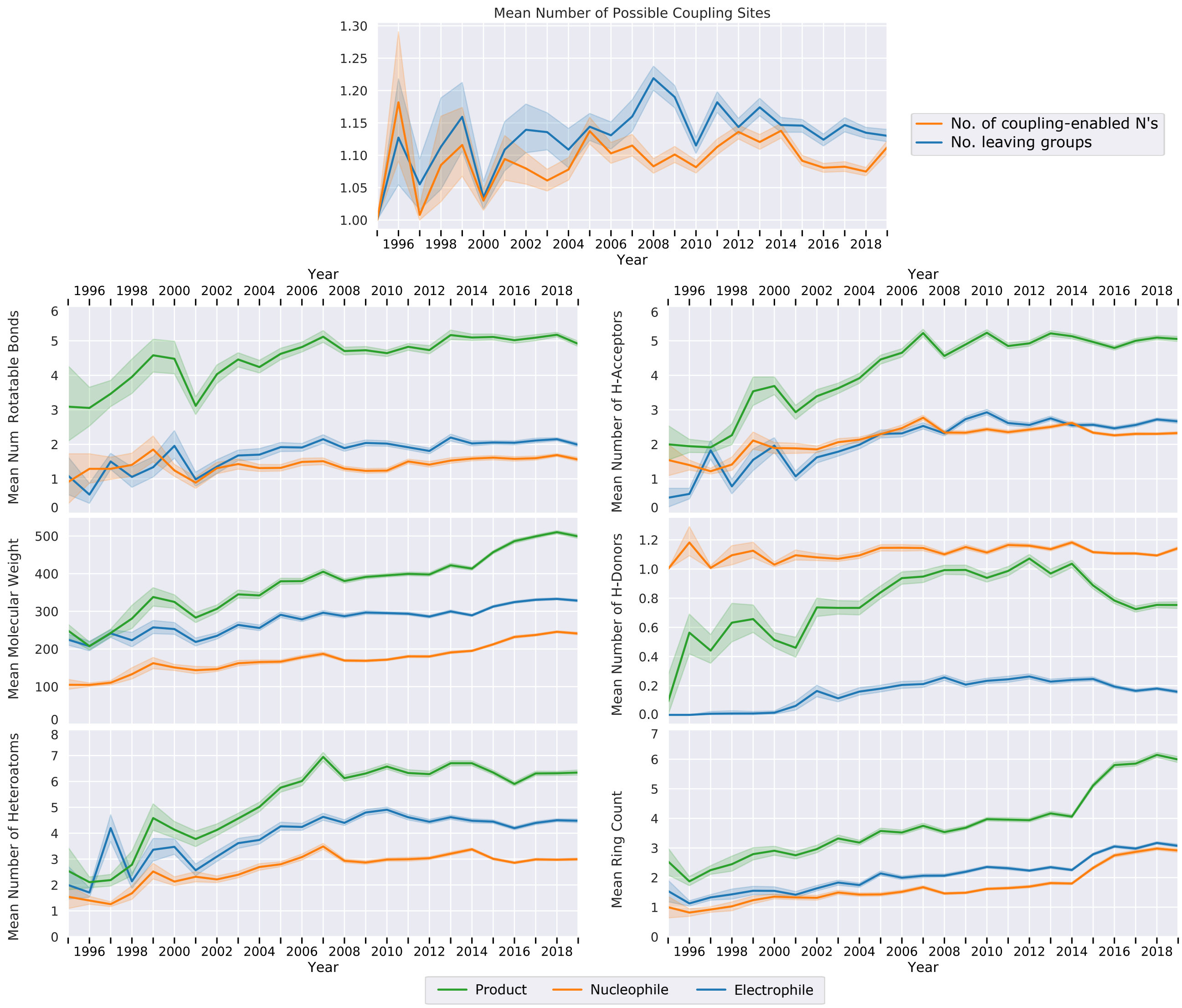

Figure S6: Time evolution of various molecular properties over time. We note for instance that i) molecular weight has steadily increased over time but the number of heteroatoms in the reactants has plateaued, indicating that in recent years molecules with more carbons are used; ii) The number of rings steadily increases, with a steep incline roughly around 2014, which coincides with the increased utilization of DiAryl and aromN substarate types.

6 Reaction Diversity Analysis

6.1 Augmented Cheat Sheet

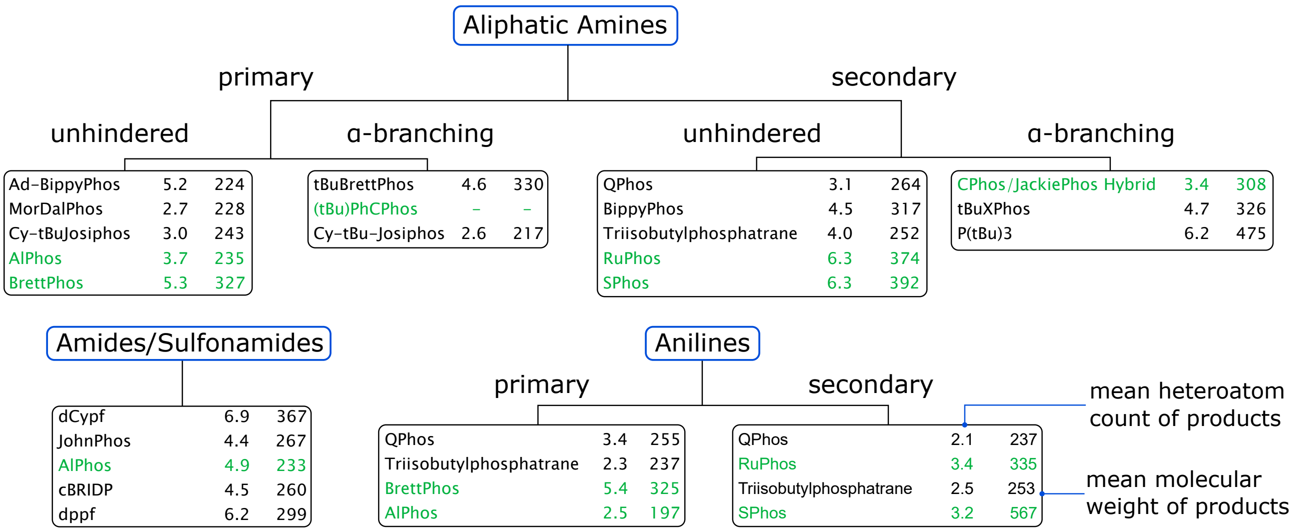

Figure S7: Copy of the cheatsheet from the main manuscript, but instead of the median yield and number of reactions it displays the mean heteroatom count and mean molecular weight of the reaction products for this nucleophile class and ligand. We see that in most cases the data-driven recommendations have similar or better metrics than the recommendations of the original cheat sheet (green). For (tBu)PhCPhos there were no entries in our data.

6.2 Nucleophile Type vs. Ligand

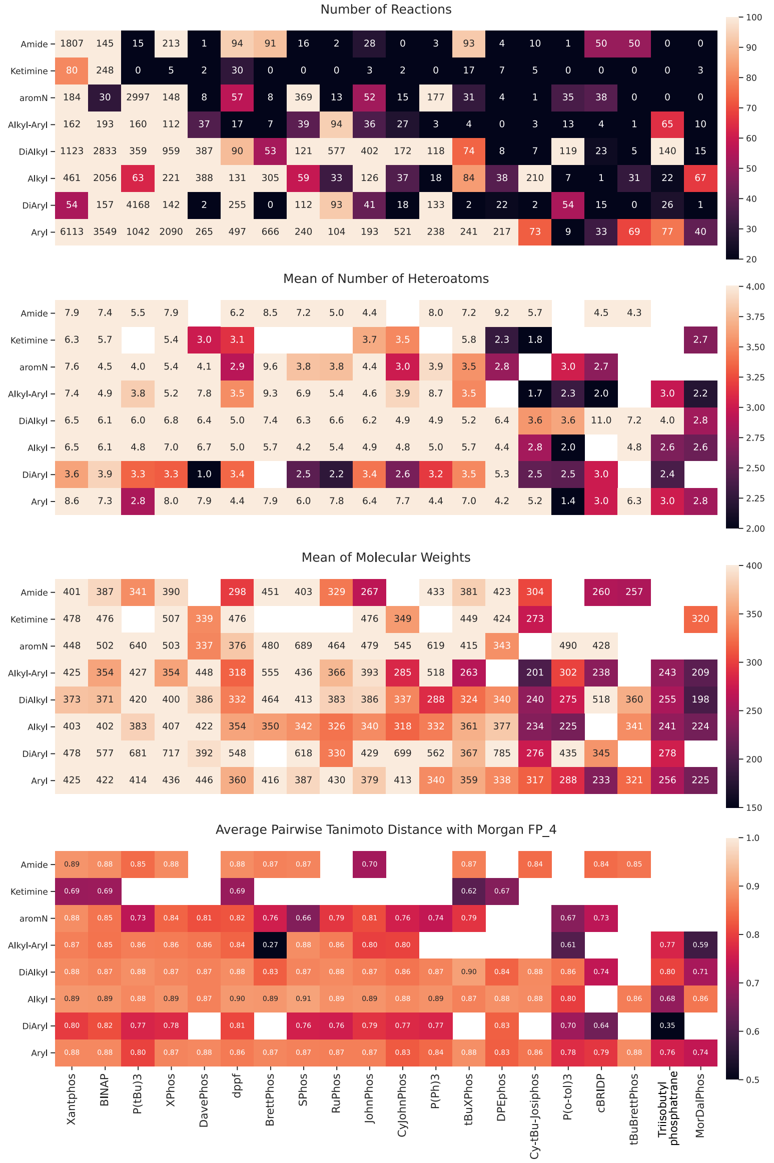

Figure S8: Diversity analysis for nucleophile class versus ligands. The top matrix shows the number of reactions for each entry while the other plots show mean properties of the reaction products.

6.3 Electrophile Type vs. Ligand

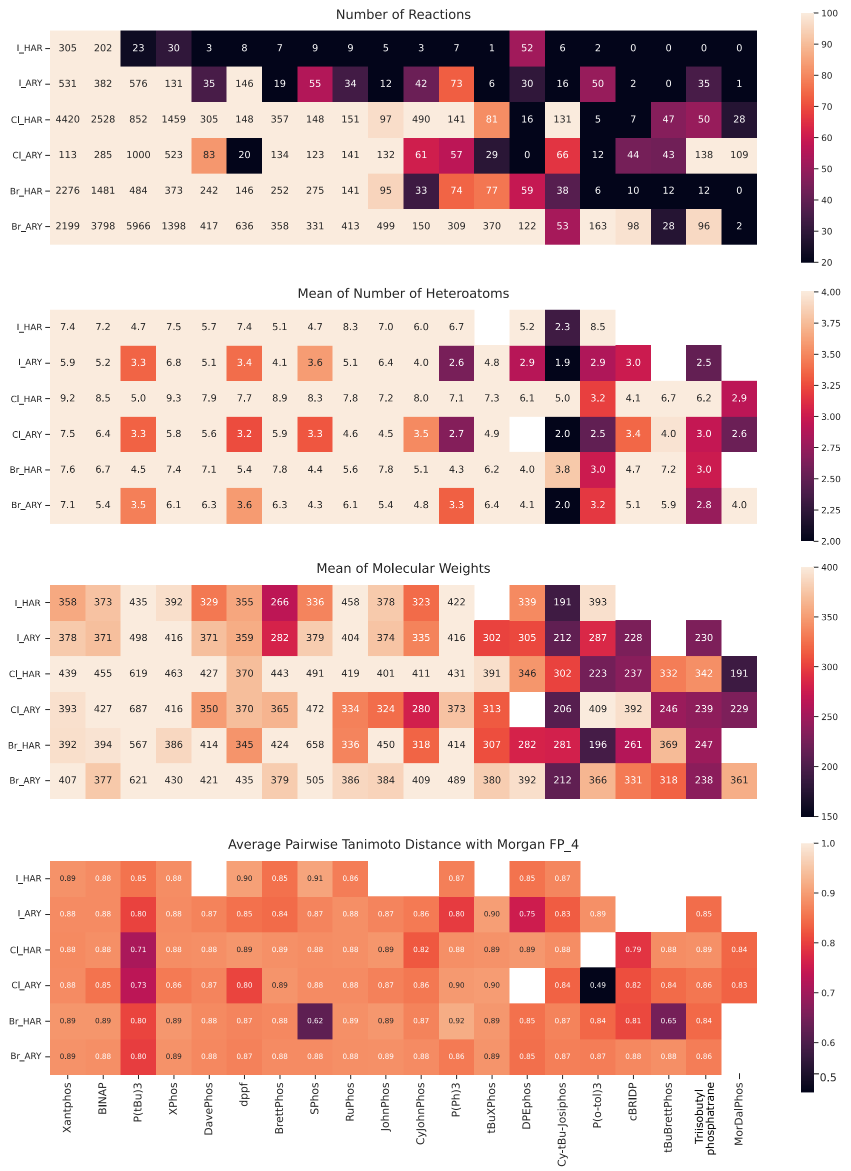

Figure S9: Diversity analysis for electrophile class versus ligands. The top matrix shows the number of reactions for each entry while the other plots show mean properties of the reaction products.

6.4 Nucleophile Type vs. Base

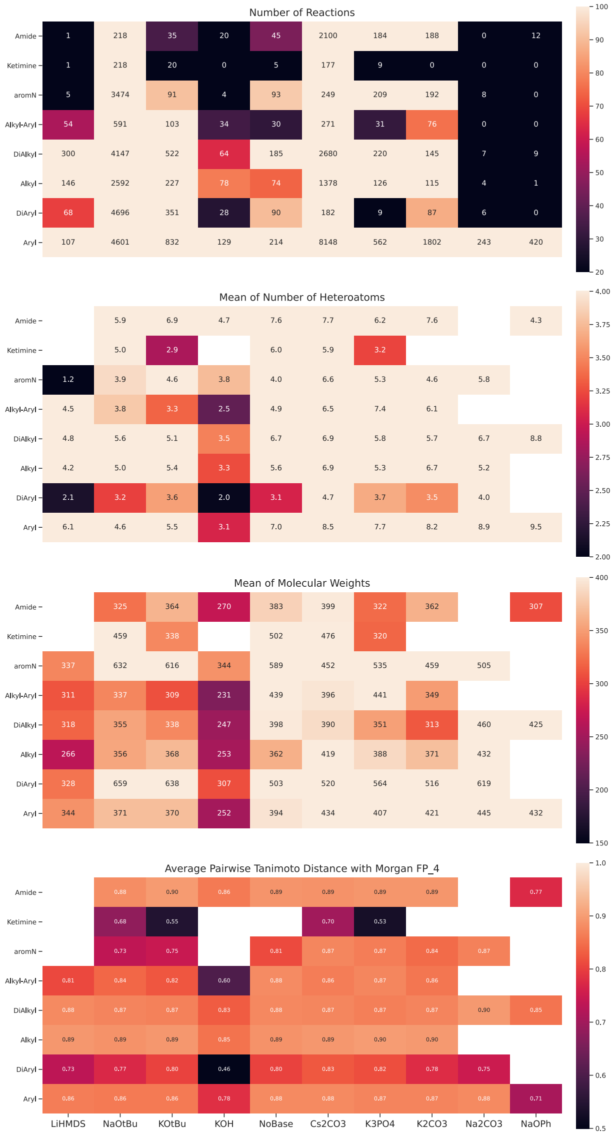

Figure S10: Diversity analysis for nucleophile class versus bases. The top matrix shows the number of reactions for each entry while the other plots show mean properties of the reaction products.

6.5 Electrophile Type vs. Base

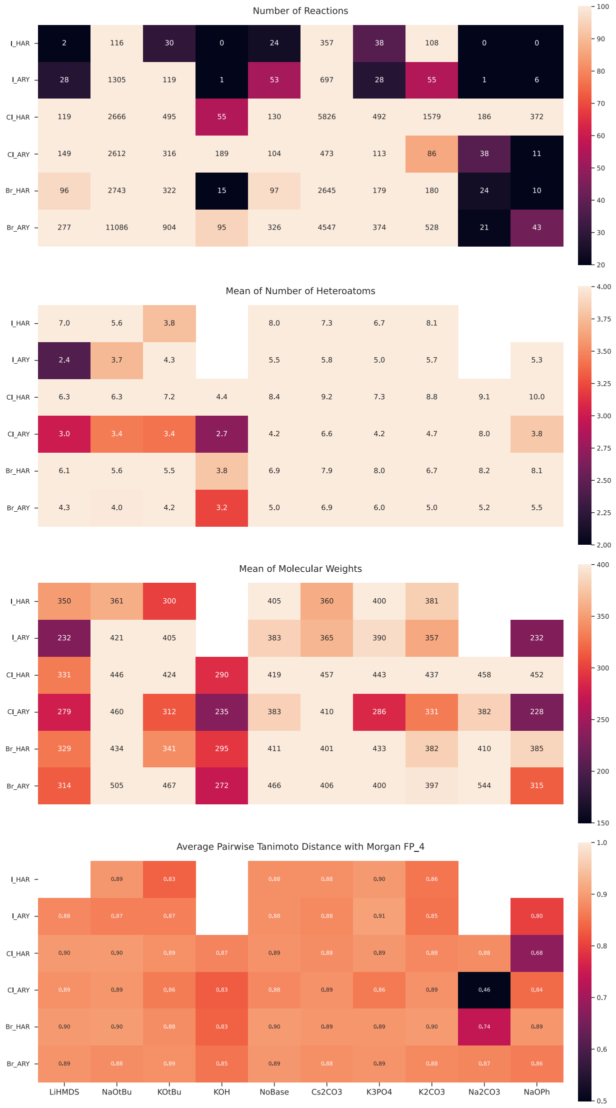

Figure S11: Diversity analysis for electrophile class versus bases. The top matrix shows the number of reactions for each entry while the other plots show mean properties of the reaction products.

7 Substance Utilization

In what follows we show an analysis of substance usage split by leaving group and substrate type. The pie charts shown indicate the fraction of publications that used the particular ligand / solvent / base. Note that not all reactions in our data record a publication. Hence, we approximate the number of publications without publication data as 1/3 of the number of reactions without publication data. This corresponds roughly to the fraction for data where publication information is available, i.e. the number of publications is 1/3 of the number of reactions.

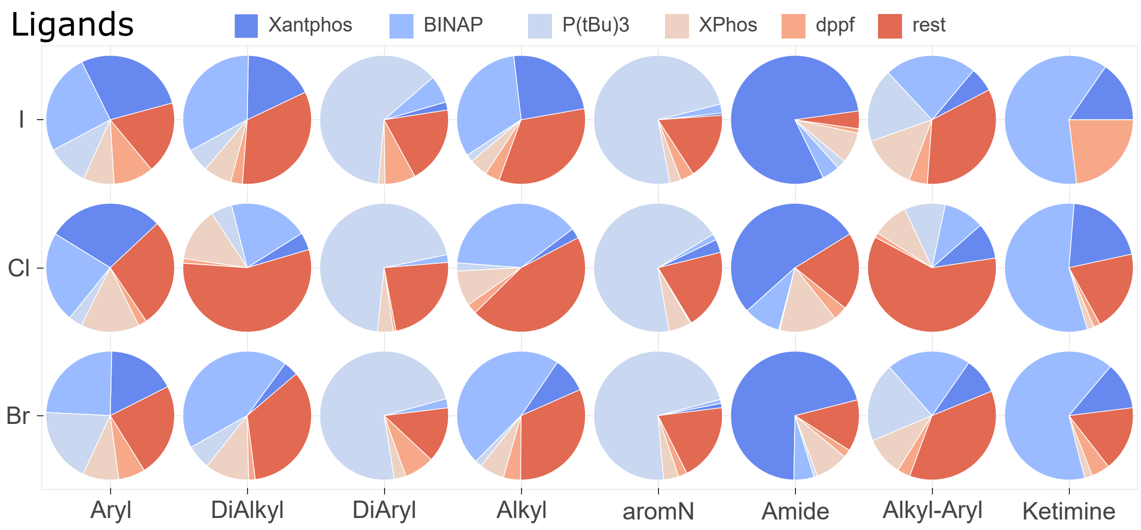

Figure S12: Utilization for the top ligands as a function of the leaving group (y axis) and substrate type (x axis). Fractions indicate publications using the corresponding substance.

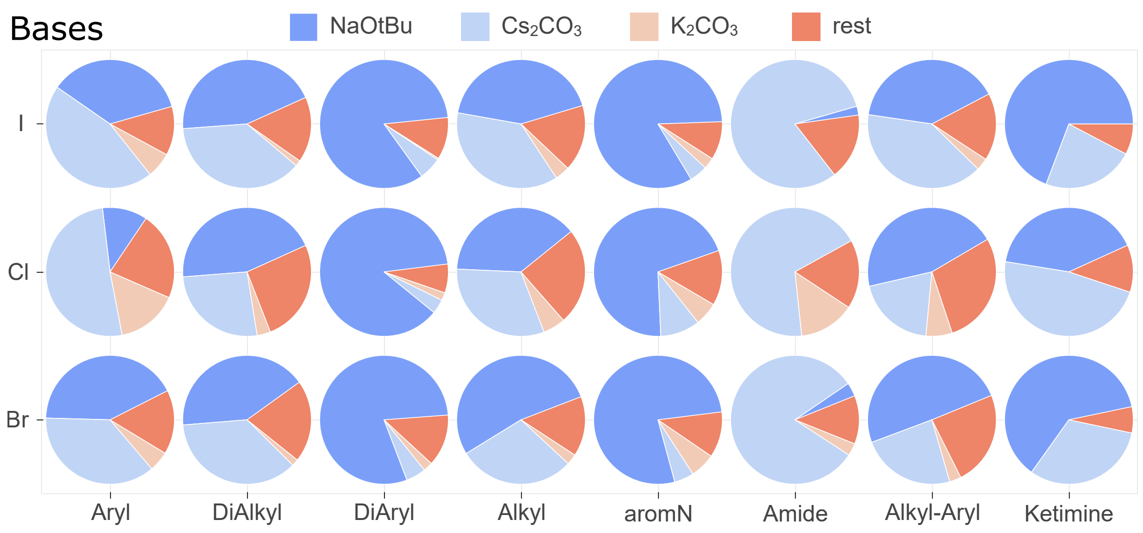

Figure S13: Utilization for the top bases as a function of the leaving group (y axis) and substrate type (x axis). Fractions indicate publications using the corresponding substance.

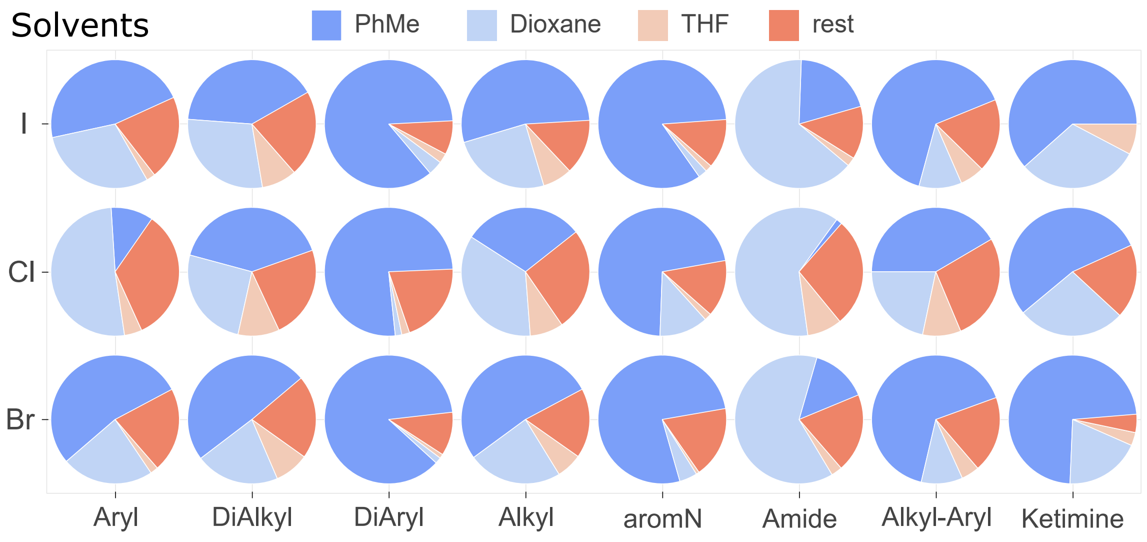

Figure S14: Utilization for the top solvents as a function of the leaving group (y axis) and substrate type (x axis). Fractions indicate publications using the corresponding substance.

8 Pre-Catalyst Analysis

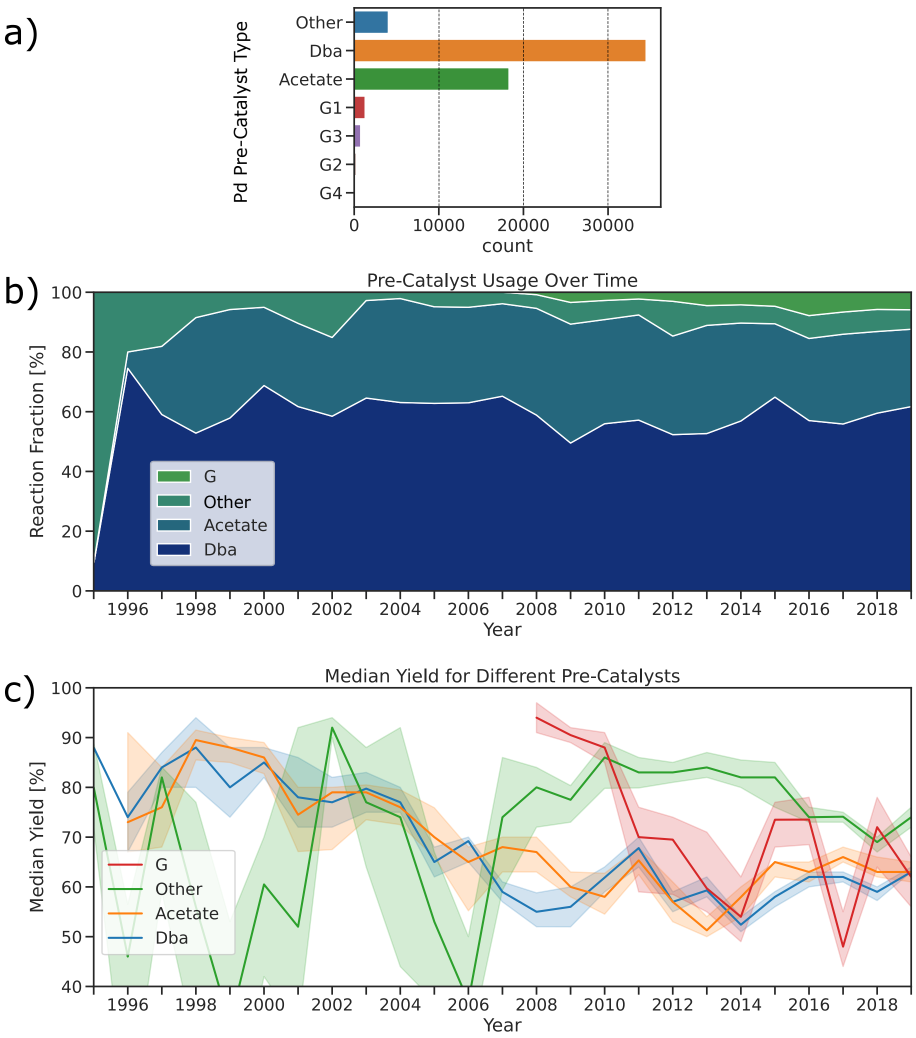

Figure S15: a) Frequency of usage for different pre-catalyst classes in our dataset. b) Time evolution of the different pre-catalyst classes. The area for each class is proportional to the percentage of reactions that utilized that class during the respective year. c) Median performance of the different pre-catalyst classes over time. The shaded are corresponds to 95% confidence intervals obtained via bootstrap resampling.

9 More Cheatsheets

In addition to the main text we provide more cheatsheets with various combinations in the file Interactive_Cheatsheet.html. The user would first decide which substance or combination of substances they want to have recommended (in the main text we show the one that is likely the most relevant, i.e. the recommendation for choosing a ligand/base combination). Then they would go to the relevant cheat sheet. Each sheet has three variants, depending on the choice of n_{Max}. The latter decides how many data points must be present at least in our data to make the respective combination appear here. It is thus a crude measure for for reliant a recommendation would be, i.e. a higher n_{Max} indicates that more data is available to back up a recommendation. However, with higher n_{Max} we see that there are not always substance combinations available with such high number of occurrences, hence a balanced choice is required. After a n_{Max} plot is chosen, the practitioner chooses the right tile by going to the one that corresponds to the electrophile type (y axis) and nucleophile type (x axis) for their relevant reaction. Finally, in the tile we show the top three recommendations based on our data, with the median yield appearing to the right. If no text or tile is printed it means there was no data available for that x-y combination.

We also provide a set of interactive matrix plots, squaring reactants vs. reagents (Interactive_ReactantsVsReagents.html), reagents vs. reagents (Interactive_ReagentsVsReagents.html), precatalysts (Interactive_Precatalysts.html) and a matrix classifying electro- and nucleophile into strongly different classes and reporting the top conditions for the combinations (Interactive_SimpleClassifications.html).

10 Ligand Ranking by Nucleophile Type

Table S1: Ligand ranking for primary Anilines. (top 50 selection)

Ligand Name	Median Yield	Yield Standard Deviation	Number of Reactions
AlPhos	98.5	0.5	2
52809-04-8	97	11.8	3
H2-IPr	96	7.2	11
776315-37-8	96	11.8	9
1246888-90-3	95	3.6	9
N-XantPhos	95	4.7	17
1105688-64-9	94.5	4.4	18
1357398-60-7	94.5	4.5	4
869336-42-5	94.5	0.8	4
97739-46-3	93	1.3	4
1157852-82-8	92.5	2.7	4
2244059-92-3	92	6.1	7
cBRIDP	92	22.9	21
869336-40-3	92	2.4	3
QPhos	91	24.3	41
DTBNpP	91	14	23
1231767-66-0	91	14.6	17
1656288-65-1	91	3.6	9
QUINAP	90	9.7	47
1126387-10-7	90	1	2
TNpP	90	10	31
MorDalPhos	90	9.8	39
1351394-95-0	90	2.5	10
TPTP	89.5	4.5	2
735272-88-5	89	2	2
1661884-13-4	89	6.2	3
SIPr	88	30.4	42
908293-83-4	88	11.9	19
1160556-62-6	88	16.5	14
1011490-00-8	87.5	2.5	2
926893-73-4	87	12.1	9
917241-97-5	87	7.1	9
dppe	86.7	9.7	2
CM-phos	86	8.3	8
Triisobutylphosphatrane	86	21.2	71
Me4tBuPhos	86	16.4	3
BippyPhos	83.5	21.6	12
1185899-00-6	83	11.5	5
BrettPhos	83	26.2	215

Table S2: **Ligand Ranking for secondary Anilines.**

Ligand Name	Median Yield	Yield Standard Deviation	Number of Reactions
1661884-13-4	99	2.8	3
714951-94-7	99	2.8	3
794527-15-4	98.5	0.5	2
SIPr	97	5.7	5
740815-37-6	96	28.9	8
255882-16-7	95.5	1.2	4
1357398-60-7	95.5	7.8	8
97739-46-3	95	1.5	15
869336-42-5	94	2.3	6
N-XantPhos	94	14.7	11
779339-46-7	93	3.2	9
QPhos	93	11.9	32
DTBNpP	93	12.9	19
94297-32-2	92	1.6	3
14185-94-5	92	15.3	11
IPr	91	37.8	12
1656288-65-1	91	3.7	9
82863-72-7	91	0.7	4
RuPhos	90.5	26.1	168
Triisobutylphosphatrane	90	17	87
1206973-44-5	90	2.3	4
1219952-94-9	89.5	22.8	12
CM-phos	89	7.7	13
1351394-95-0	89	4.7	5
Triethylphosphine	87.5	3.5	2
DavePhos	87	24.8	39
2242044-02-4	86	14	2
1246888-90-3	86	8	2
Di-tert-butylphosphine	86	5.8	3
cBRIDP	85	23.6	19
384842-24-4	84.5	27.6	6
6476-36-4	81.5	3.3	4
Tris(tert-butoxy)phosphine	81	5.7	6
H2-IPr	81	4.5	3
CataCXium A	80	12.2	7
P(Cy)3	78.5	19.7	22
JohnPhos	77	21.9	77
2064140-39-0	73	6	2
AmPhos	73	14.9	18
1011490-00-8	72.5	2.5	2
P(Ph)3	72.4	14.9	134
P(tBu)3	72	15.3	4315
MorDalPhos	71	9.5	11
CyJohnPhos	69.9	27.3	45
XPhos	68	20.4	238
BINAP	68	22.7	318
142691-72-3	68	5	2
dppf	66.6	17.6	267

Table S3: Ligand ranking for primary, unhindered Alkyls.

Ligand Name	Median Yield	Yield Standard Deviation	Number of Reactions
1450877-22-1	96	5.6	5
255882-16-7	93.5	5.5	2
Ad-BippyPhos	93	5.9	61
740815-37-6	93	3.2	6
QPhos	93	5.2	17
1126387-10-7	93	0	2
779339-46-7	91.5	4.6	4
tBuBrettPhos	91	19	5
908293-83-4	91	10.1	12
628333-86-8	89.5	0.5	2
64741-27-1	88	10	2
H2-IPr	87	10.1	21
1242030-74-5	87	20	5
1661884-13-4	85.5	4.5	2
MorDalPhos	85	7.8	50
894085-97-3	84.5	5.5	2
AlPhos	83.5	10.4	6
1656288-65-1	83.5	0.5	2
Cy-tBu-Josiphos	83	20.3	135
CM-phos	82	5.1	3
1262046-33-2	81.5	7.5	2
BippyPhos	81.5	30.6	26
1166994-77-9	81	29	5
2064140-39-0	80	15.7	3
950982-50-0	80	14.9	18
142691-72-3	80	7.2	4
259660-18-9	79	17.7	11
DPEphos	78.5	21.4	16
QUINAP	73.5	15.3	4
1246888-90-3	73.5	1.5	2
1156461-30-1	73	0	2
CyPFt-Bu	73	13.9	3
RuPhos	72.5	25.6	16
dppp	72	22.5	9
415941-58-1	71.5	8.2	4
672937-62-1	70	11.6	7
BrettPhos	68	25.2	154
Triisobutylphosphatrane	68	0	4
P(o-tol)3	68	25.1	5
JohnPhos	64	27.6	77
1231767-66-0	63	19.2	9
PPFt-Bu	62.5	31.4	6
dppf	62	22.3	81
BINAP	61	25.7	852
tBuXPhos	60.6	27.8	28
DTBPF	59.1	32.1	2
264284-69-7	59	28.4	9
Me4tBuPhos	57	17.4	4

Table S4: Ligand ranking for primary, α-branching Alkyls.

Ligand Name	Median Yield	Yield Standard Deviation	Number of Reactions
894085-97-3	99	0	2
CyPFt-Bu	88.5	2.5	2
908293-83-4	88	7.6	27
tBuBrettPhos	88	22.3	25
Ad-BippyPhos	87.5	7.3	8
1447963-71-4	85	25.3	11
2244059-92-3	84	8	2
(tBu)PhCPhos	82	8.9	19
1656288-65-1	80.5	0.5	2
H2-IPr	80	10.5	5
CM-phos	75	8.6	8
Cy-tBu-Josiphos	74.5	24.6	70
1450877-22-1	74	28.3	4
MorDalPhos	74	11.5	17
p-tolyl-BINAP	72	16.8	13
CataCXium A	72	22	2
RuPhos	71	26.8	17
tBuXPhos	70.5	26.4	56
IPr	70	17.4	3
BippyPhos	70	20.2	25
QPhos	68.5	25.6	10
Triisobutylphosphatrane	67	22.9	18
dppf	65.5	25.5	50
QUINAP	61.5	1.5	2
1156461-30-1	60	8.5	3
JohnPhos	57	23.6	49
P(tBu)3	57	20.4	42
BINAP	57	24.4	1182
BrettPhos	56	27.5	149
dppp	56	22.2	10
DPEphos	55.5	17.1	22
SPhos	54.5	22	34
Xantphos	51.2	24.3	206
P(o-tol)3	50.6	5.4	2
XPhos	40.4	23.9	74
DavePhos	39	24.9	303
264284-69-7	30	15	5
CyJohnPhos	26.1	30	14
AmPhos	20.2	0.5	2
P(Ph)3	19	31.2	11
142691-72-3	15	2	2
dCypf	12	1	2

Table S5: Ligand ranking for secondary, unhindered Alkyls.

Ligand Name	Median Yield	Yield Standard Deviation	Number of Reactions
2144425-53-4	99	15.5	6
1661884-13-4	99	5.2	3
97739-46-3	96.5	7.5	18
894085-97-3	96	1.3	5
1504583-87-2	94	6.4	4
1231767-66-0	93	30.6	7
QPhos	93	20.9	23
2244059-92-3	93	11.3	15
794527-15-4	92.5	6.5	2
94297-32-2	92.5	4.3	4
735272-88-5	92	16	4
779339-46-7	91.5	4.8	8
DTBNpP	91	6.9	15
255882-16-7	91	6	7
BippyPhos	90.5	29	16
1246888-90-3	90.5	8.5	2
1656288-65-1	90	5.2	15
1156461-30-1	90	8.5	3
714951-94-7	90	13.3	5
64741-27-1	90	12.8	3
628333-86-8	90	4.1	5
1450877-22-1	90	6.9	5
338799-92-1	88.5	4.5	2
H2-IPr	88.5	23.7	24
869336-42-5	88	7.8	3
1126387-10-7	88	13.8	7
dppb	87	40.1	3
1357398-60-7	86	8.9	4
740815-37-6	86	11	2
1351394-95-0	85	3.8	9
CM-phos	84	9.1	19
AmPhos	83.5	7.3	6
1242030-74-5	83	14.5	11
Triisobutylphosphatrane	82.5	17.2	138
N-XantPhos	82	9.8	6
SIPr	81	21.1	47
IPr	81	27.7	31
PEPFINE	81	3	2
2242044-02-4	80	5	2
CataCXium A	80	27	23
879904-89-9	78	13.7	3
735272-79-4	77	19	2
405877-65-8	77	22.3	5
82863-72-7	77	25.8	10
Cy-tBu-Josiphos	76	7.3	7
1046119-14-5	75.5	12.9	6
1352128-71-2	75.5	15.4	4
P(Ph)3	74.1	25.6	98

Table S6: Ligand ranking for secondary, α-branching Alkyls.

Ligand Name	Median Yield	Yield Standard Deviation	Number of Reactions
JackiePhos	92	4.8	3
dppb	92	2	2
1021176-69-1	85	15.1	7
1810068-30-4	81	13.5	9
H2-IPr	79.5	9.6	4
tBuXPhos	76	18.5	24
IPr	75	19.4	5
P(tBu)3	65	28.4	71
SPhos	59	29.2	9
DTBPF	58.5	16.5	2
dppf	55	31.1	5
BINAP	52.5	22.7	220
BippyPhos	49.5	41.5	2
Xantphos	47.4	24.2	76
DavePhos	46	27.3	32
RuPhos	38	28.9	75
Triisobutylphosphatrane	32.1	11.5	2
CyJohnPhos	31.9	18.1	7
XPhos	31.2	25.4	57
JohnPhos	30.5	13.8	16
P(Ph)3	30.5	20.7	20
P(o-tol)3	22.7	28.2	7
1360762-07-7	13	14.2	9
SIPr	12.5	7.5	2
BrettPhos	7	7.2	4

Table S7: Ligand ranking for Amides / Sulfonamides.

Ligand Name	Median Yield	Yield Standard Deviation	Number of Reactions
dCypf	96	10.7	54
1345160-30-6	94	10.4	12
1083181-51-4	93	3	2
594815-59-5	93	6.7	6
QPhos	87.5	1.5	2
JohnPhos	87.5	26.7	28
AlPhos	86	14.6	8
908293-83-4	85	13.5	27
cBRIDP	85	23	50
P(Ph)3	84	5.5	3
tBuBrettPhos	84	13.9	52
dppf	84	23	95
DavePhos	83.5	9.5	2
BippyPhos	82	22.9	32
856405-77-1	82	13.4	4
Cy-tBu-Josiphos	81.5	34	8
P(tBu)3	80	14	15
DTBPF	78.4	14	4
JackiePhos	77	23	11
RuPhos	76.5	16.5	2
Triisobutylphosphatrane	32.1	11.5	2
tBuXPhos	69	22.4	120
BINAP	69	25	146
dppp	68	21	2
Me4tBuPhos	66	27	34
Xantphos	62	25.2	1831
XPhos	62	25.5	223
SPhos	55.2	34.4	16
CyPFt-Bu	55	44	2
BrettPhos	52	22.9	93
a 1244949-53-8	50.8	31	2
DPEphos	39	24	4
50595-38-5	17	8	2
